# Epidemiology of leisure-time physical activity in socio-demographic, lifestyle and psychological characteristics of men and women in Greece: the ATTICA Study

**DOI:** 10.1186/1471-2458-5-37

**Published:** 2005-04-18

**Authors:** Christos Pitsavos, Demosthenes B Panagiotakos, Yannis Lentzas, Christodoulos Stefanadis

**Affiliations:** 1 First Cardiology Clinic, School of Medicine, University of Athens, Athens, Greece; 2 Department of Nutrition and Dietetics, Harokopio University, Athens, Greece

## Abstract

**Background:**

We aimed to evaluate the prevalence, frequency and type of leisure-time physical activity (LTPA) among adults in Greece, as well as its relationship with socio-demographic, lifestyle and clinical characteristics of these people.

**Methods:**

From May 2001 to December 2002 we randomly enrolled 1514 men and 1528 women, without any evidence of cardiovascular or any other chronic disease. The sampling was stratified by the age – gender distribution of (census 2001) of the greater area of Athens. Weekly energy expenditure assessed by considering frequency, duration (in minutes) and intensity of sports related physical activity during a usual week.

**Results:**

53% of men and 48% of women were classified as physically active. Men were more likely to be active as compared to women (p < 0.05), while the lowest activity rates were observed in 40 to 49 years old participants (p < 0.01). Physically active people had higher occupation skills, were more likely to live in rural areas, to be unmarried, non smokers and they were devoted to a healthier dietary pattern, as compared to sedentary, irrespective of age and sex (all p < 0.05). In addition, the cumulative risk factors score of obesity, hypertension, hypercholesterolemia and diabetes, was inversely associated with activity status (p < 0.001). Finally, physically active men and women were less likely to report depressive symptoms (p < 0.01), after various adjustments were made.

**Conclusion:**

Half of the studied population reported physically inactive, indicating that sedentary lifestyle becomes a serious epidemic in Greece. High occupation skills, non-smoking, devotion to a healthier dietary pattern and a better cardiovascular risk factors profile were some of the determinants of physically active people.

## Background

Several observational and clinical studies suggest that physical activity substantially reduces the risk of dying of coronary heart disease, stroke, and colon cancer [1-4]. It also helps to control weight, contributes to healthy bones, muscles, and joints, reduces falls among older adults, helps to relieve the pain of arthritis, reduces symptoms of anxiety and depression and is associated with fewer hospitalizations, physician visits, and medications [3]. Despite the proven benefits of physical activity, the National Center for Chronic Disease Prevention and Health Promotion reports that more than one half of American adults do not get enough physical activity to provide health benefits, while 25% of adults are not active at all in their leisure time [3]. Worldwide, the World Health Organization estimates that over 60% of adults are not active enough to benefit their health [4]. Moreover, physical activity declines significantly with age, it is generally higher among females, and the overall inactivity trend is worse in poor urban areas. In addition, there are racial and ethnic differences in physical activity rates, particularly among women.

Data regarding the prevalence of physical activity and its association with various characteristics of Greek men and women are lacking. Therefore, the aim of this work is to evaluate the prevalence of leisure-time physical activity (LTPA) and to investigate its association with various socio-demographic, lifestyle and behavioural characteristics of Greek adults.

## Methods

The "ATTICA" study is a health and nutrition survey, carried out in the province of Attica (including 78% urban and 22% rural areas), where Athens, is a major metropolis. The sampling was random, multistage and based on the city – gender – age distribution of the province of Attica (census of 2001). The study's design anticipates enrolling only one participant per household. The main goals of the ATTICA study were to record the distribution of several blood lipids, inflammatory, oxidation, coagulation, thrombotic and clinical factors and to explore the associations between these factors with several socio-demographic, lifestyle and psychological characteristics of the participants.

### Study's participants

From May 2001 to August 2002, 4056 inhabitants from the above area were randomly asked to participate in the study. Of those, 3042 agreed to participate (75% participation rate); 1514 of the participants were men (50%, 20–87 years old) and 1528 were women (50%, 20–89 years old). Between 40 – 49 years old there were 48% men and 52% women. The number of the participants was determined by power analysis and chosen to evaluate two-sided differences between the normally distributed investigated parameters and physical activity groups greater than 10%, achieving statistical power > 0.80 at < 0.05 probability level (*P*-value). Also, those living in institutions were excluded from the sampling. The participants had no clinical evidence of cardiovascular or any other atherosclerotic disease, as well as chronic viral infections dental problems or any type of surgery in the past week. They also exhibited no signs of cold, flu, or any acute respiratory infection.

### Physical activity ascertainment

We used a translated version of a validated questionnaire [5] of weekly energy expenditure that assessed: frequency (times per week), duration (in minutes) and intensity of sports related physical activity during a usual week. Intensity was graded in qualitative terms such as: light (expended calories < 4 Kcal/min, i.e. walking slowly, cycling stationary, light stretching etc.), moderate (expended calories 4–7 Kcal/min, i.e. walking briskly, cycling outdoor, swimming moderate effort etc.) and high (expended calories >7 Kcal/min, i.e. walking briskly uphill, long distance running, cycling fast or racing, swimming fast crawl etc.). Participants who did not report any physical activities were defined as sedentary. For the rest, we calculated a combined score by multiplying the weekly frequency, duration and intensity of physical activity. Then we calculated the tertiles of this score, and physically active participants were equally classified into three groups: low physical activity (1^st ^tertile), medium physical activity (2^nd ^tertile) and high physical activity (3^rd ^tertile). Therefore, 1^st^, 2^nd ^and 3^rd ^tertile included 217 men and 205 women, respectively. We have also recorded the type of exercise, i.e. resistance (i.e. any technique that uses progressive resistance to increase muscular strength) or endurance training. The presence of occupational physical activity was recorded (41% of men and 27% of women reported occupational physical activity at least one time per week, i.e. mainly manual workers like builders, mason, labourer, technicians, etc), but was not taken into account for the analysis due to difficulties in evaluation and standardization. This exclusion may confound our findings, but the large sample size and the randomised procedure for the selection of the participants can spread the subjects who reported occupational exercise equally in both groups of the study.

Although there are some concerns with self-reported physical activity, the introduced evaluation is now considered reliable, valid and has been used by many other similar studies [6].

### Socio-demographic and behavioural characteristics

In addition to the physical activity status, the study's questionnaire included demographic characteristics like age, gender, family status (married, divorced, widowed), financial status (average annual income during the past three years), and occupational status as well as education level. The educational level of the participants (as a proxy of social status) was measured in years of school. Occupation was also recorded and evaluated through a ten-point scale from unskilled – hand workers (lower values) to executive – skilled workers (higher values).

Information about smoking habits was collected using a standardized questionnaire developed for the Study. Current smokers were defined as those who smoked at least one cigarette per day. Never smokers those who have never tried a cigarette in their life and former smokers were defined as those who had stopped smoking more than one year previously. In all multivariate statistical analyses cigarette smoking habits were taken into account using the pack-years (cigarette packs per day × years of smoking). However, in order to correct for the amount of nicotine containment in various types of cigarettes smoked (i.e. light, heavy, very heavy), we assigned a weight in each different type of cigarette – pack, using the 0.8 mg/cigarette as the standard.

Consumption of non-refined cereals and products, vegetables, legumes, fruits, olive oil, dairy products, fish, pulses, nuts, potatoes, eggs, sweets, poultry, red meat and meat products were measured as an average per week during the past year through a validated food – frequency questionnaire (FFQ), from the Department of Nutritional Epidemiology of Athens Medical School [7]. The frequency of consumption was then quantified approximately in terms of the number of times a month a food was consumed. Alcohol consumption was measured by daily ethanol intake, in wineglasses (100 ml and 12 gr ethanol concentration). Based on the Mediterranean – diet pyramid [8], we calculated a special diet score ranged from 0 to 55. Higher values of this score indicates adherence to the traditional Mediterranean diet, characterized by moderate consumption of fat and high monounsaturated: saturated fat ratio), while lower values indicate adherence to the "Westernized" diet.

Depressive symptomatology was assessed using a translated and validated version of the Zung Self-Rating Depression Scale (ZDRS) [9]. The ZDRS is a well known and world-widely used self-rating scale for the measurement of depression. It is a self-reporting instrument and was originally developed in order to assess depression symptoms without the bias of an administrator affecting the results. Higher scores on this scale are indicative of more severe depression [9]. ZDRS consists of 20 items that cover affective, psychological, and somatic symptoms. The patient specifies the frequency with which the symptom is experienced (that is: a little = 1, some = 2, a good part of the time = 3, or most of the time = 4). A subject with ZDRS score below 50 is considered normal, with a score of 50–59 is considered to suffer from mild depression, with score 60–69 depression is considered moderate, while with a score of 70 or above depression is considered to be severe [9]. Previous investigations indicate that the ZDRS is a valid and sensitive measure of clinical severity in depressed patients and support its continued use as a research instrument [10]. Moreover, the sensitivity of the ZDRS was found adequate, and the scale was able to significantly differentiate four severity groups classified on the basis of the global rating.

### Biochemical and clinical measurements

The blood samples were collected from the antecubital vein between 8 to 10 a.m., in a sitting position after 12 hours of fasting and avoiding of alcohol. The biochemical evaluation was carried out in the same laboratory that followed the criteria of the World Health Organization Lipid Reference Laboratories. Serum cholesterol was measured using chromatographic enzymic method in a Technicon automatic analyzer RA-1000. The intra and inter-assay coefficients of variation of cholesterol levels did not exceed 5%. Participants with total serum cholesterol levels greater than 200 mg/dl or those taking lipid-lowering agents were classified as hypercholesterolemic and those with blood sugar > 125 mg/dl or the use of antidiabetic medication were classified as diabetic. Arterial blood pressure was measured at end of the physical examination with subject in sitting position for 25–30 minutes. Three measurements were abtained from the right arm (*ELKA using aneroid manometric sphygmometer*, *Von Schlieben Co*, *West Germany*). Patients whose average blood pressure levels were greater or equal to 140/90 mm Hg or taking antihypertensive medication were classified as hypertensives. Body mass index was calculated as weight (in kilograms) divided by standing height (in meters squared). Obesity was defined as body mass index > 29.9 kg/m^2^. Also, the investigators of the study recorded a detailed medical history of the participants.

Details about the aims and methods of the ATTICA study have been presented elsewhere [11].

### Statistical analysis

Continuous variables are presented as mean values ± standard deviation, while categorical variables are presented as absolute and relative frequencies. Associations between categorical variables were tested by the use of contingency tables and the calculation of chi-squared test. Correlations (or partial correlations) between continuous or discrete variables were tested by the calculation of Pearson's *r *or Spermans's *rho *(for skewed variables) correlation coefficients. Comparisons between normally distributed continuous variables and categorical were performed by the use of Analysis of co-Variance (multi- way ANCOVA), after controlling for homoscedacity, and potential confounders. Kolmogorov-Smirnov criterion was used for the assessment of normality. In the case of asymmetric continuous variables the tested hypotheses were based on the calculation of the Kruskal – Wallis test. Associations between the investigated variables and physical activity levels were tested through fixed effects models, after the adjustment for several potential confounders and its interactions with physical activity status. The assumptions of linearity and homoscedacity were graphically tested by plotting fitted values against standardised residuals. Multiple logistic regression analysis estimated the odds ratio of being at a specific health condition with respect to physical activity status. Deviance residuals evaluated models' goodness of fit. All reported *P*-values are based on two-sided tests and compared to a significance level of 5%. However, due to multiple significance comparisons we used the Bonferronni correction in order to account for the increase in Type I error. SPSS 11.0.5 software (SPSS Inc. 2002, Texas, USA) was used for all the statistical calculations.

## Results

Eight hundred and two (53%) of men and 733 (48%) of women were classified as physically active. Table 1 presents the age distribution of physical activity in study's participants. Men were more likely to be physically active as compared to women, across all age groups (p = 0.001). The lowest physical activity rates were observed between 40 to 49 years old men and women (p = 0.01). Physically active men were devoted to more intense activities as compared to women (6.96 ± 1.2 vs. 6.23 ± 1.1 kcal/min, p = 0.02). In addition, men used to exercise 3.2 ± 1.7 times per week (for 85 ± 19 min/time) and women 2.7 ± 1.3 times per week (for 42 ± 21 min/time) (p = 0.03). An inverse relationship was observed between age and frequency, intention and time devoted to physical activities (partial *r *= -0.23, p = 0.02, *r *= -0.34, p = 0.001, and *r *= -0.21, p = 0.0001, respectively).

**Table 1:** Distribution of physical activity by age and sex

	Physically active	
Age group (men / women)	Men (n = 1514)	Women (n = 1528)
20 – 29 y	52.2%	48.0%
30 – 39 y	43.1%	41.0%
40 – 49 y	37.1%	35.1%
50 – 59 y	47.3%	37.5%
> 60 y	45.4%	38.2%

Furthermore, from physically active participants, 5% of men and 2% of women reported only resistance training, while 7% of men and 12% of women reported only endurance training. Thirty five percent of men and 25% of women reported that they were engaged in organised sports (sport clubs, or football, basketball and volleyball teams, etc.). When we compared participants in organised sports with non-participants, those who participated in organised sports were more often persistent exercisers as compared to those who did not (odds ratio = 11.6 for men, 95% confidence interval 7.2 to 15.1 and 7.9 for women, 95% confidence interval 5.5 to 11.0).

### Physical activity and socio-demographic characteristics

Table 2 presents social characteristics of the participants by physical activity status. In particular, a positive association was observed between physical activity and occupation skills (unskilled to skilled) in men and in women (both p < 0.05). Moreover, people living in rural areas were more likely to be physically active as compared to people living in urban areas (55% vs. 46%, p = 0.02). Never married participants were more likely to be physically active as compared to married or divorced/widowed (57% vs. 44%, p < 0.001), irrespective of age and sex. No statistically significant associations were found between physical activity levels and other social status indices (education level and annual income).

**Table 2:** Socio – demographic characteristics by physical activity status and sex

	*Physical activity status*				
*Men*	*Sedentary*	*1*^*st*^*tertile*	*2*^*nd*^*tertile*	*3*^*rd*^*tertile*	*P*
Education (years of school)	12 ± 4	12 ± 4	13 ± 4	12 ± 5	0.18
Annual income (× 1000 Euros)	18,2 ± 3	16,9 ± 3	16,2 ± 5	15,8 ± 5	0.23
Occupational status^⊥ ^(0 – 10)	5,1 ± 2	6,1 ± 3*	6,2 ± 3*	7,0 ± 5**	0.02
*Women*	*Sedentary*	*1*^*st*^*tertile*	*2*^*nd*^*tertile*	*3*^*rd*^*tertile*	*P*
Education (years of school)	11 ± 4	11 ± 3	13 ± 5	12 ± 2	0.36
Annual income (× 1000 Euros)	15,4 ± 7	15,8 ± 7	17,7 ± 6	14,2 ± 6	0.35
Occupational status^⊥ ^(0 – 10)	4,9 ± 2	6,5 ± 3**	6,9 ± 3*	7,8 ± 3**	0.01

Variables are expressed as mean value ± SD

^⊥^Occupational status is measured in a ten-unit scale where lower values imply unskilled professions and higher values skilled.

*P*-values of the last column derived from stratified ANCOVA tests, after controlling for age and denote the significance of the association between physical activity status and education, income and occupational status of the participants.

* *P *< 0.05 and ** *P *< 0.01 Bonferronni corrected probability values for the comparisons between physical activity groups and sedentary participants.

### Physical activity, lifestyle and behavioural characteristics

Table 3 illustrates the associations between physical activity and various lifestyle and behavioural characteristics of the participants. Particularly, we observed that physically active participants were less frequent to be active smokers as compared to sedentary. An inverse relationship was found between physical activity and dietary score, which implies that physically active participants showed greater adherence to a healthier dietary pattern, i.e. the Mediterranean diet (higher values of the diet score). In addition, active men and women consumed lower quantities of alcoholic beverages as compared to sedentary. Multivariate analysis (that used physical activity index as an independent variable and diet score as a dependent variable) showed that all the previous associations were independent from age, sex, education and financial status of the participants, as well as region of living (all p's < 0.05).

**Table 3:** Behavioural characteristics by physical activity status and sex

	*Physical activity status*				
*Men*	*Sedentary*	*1*^*st*^*tertile*	*2*^*nd*^*tertile*	*3*^*rd*^*tertile*	*P*
Current smoking	58%	54%	54%	48%*	0.01
Alcohol intake (ml/d)	280 ± 40	250 ± 55*	190 ± 95**	180 ± 90**	0.001
Diet score (0 – 55)	28 ± 10	32 ± 25*	38 ± 23**	41 ± 22**	0.001
ZDRS (0 – 100)	48 ± 23	41 ± 23	38 ± 16	32 ± 17	< 0.001
*Women*	*Sedentary*	*1*^*st*^*tertile*	*2*^*nd*^*tertile*	*3*^*rd*^*tertile*	
Current smoking	49%	44%	36%**	29%**	0.041
Alcohol intake (ml/d)	120 ± 40	110 ± 55	90 ± 55*	85 ± 50**	0.001
Diet score (0 – 55)	29 ± 11	36 ± 22*	42 ± 19**	43 ± 21**	< 0.001
ZDRS (0 – 100)	52 ± 21	44 ± 13*	39 ± 13**	34 ± 19**	0.001

Variables are expressed as mean value ± SD or relative frequencies.

*P*-values of the last column derived from stratified ANCOVA tests, after controlling for age and denote the significance of the association between physical activity status and smoking, dietary habits and depression status of the participants.

* *P *< 0.05 and ** *P *< 0.01 Bonferronni corrected probability values for the comparisons between physical activity groups and sedentary participants.

An inverse relationship was observed between physical activity status and ZDRS, which indicates that men and women who used to exercise were less likely to have depressive symptoms as compared to sedentary (standardised beta-coefficient = -0.34, p = < 0.001), irrespective from the effect of various potential confounding factors like age, sex, educational, financial and occupational status. Moreover, participants in the highest tertile of physical activity had 41% lower odds (95% confidence interval 0.35 to 0.9) of having major depressive syndromes (i.e. ZDRS > 60), after controlling for the previous potential confounding factors.

### Physical activity and clinical characteristics

An inverse relationship was observed between body mass index and physical activity score (partial *r *= -0.46, p < 0.001), after adjusting for age and sex. Participants in the highest tertile of physical activity had 48% lower odds of being obese (95% confidence interval 0.42 to 0.64), after adjusting for age, sex, smoking habits, blood pressure, total cholesterol and glucose levels. The prevalence of hypertension was 38% in men and 24% in women. Physical activity was also associated with lower odds of being hypertensive (odds ratio = 0.76, 95% confidence interval 0.59 to 0.98), irrespective of age, sex, smoking habits, total cholesterol and glucose levels. Moreover, compared to sedentary, people in the highest tertile of physical activity had on average 15 mm Hg lower systolic (p = 0.02) and 8 mmHg lower diastolic (p = 0.04) blood pressure levels, after adjusting for age, sex, body mass index and diet score. We have also observed that 54% of men and 60% of women had total serum cholesterol levels <200 mg/dl. No association was observed between physical activity and presence of hypercholesterolemia (p = 0.76).

**Figure 1:**
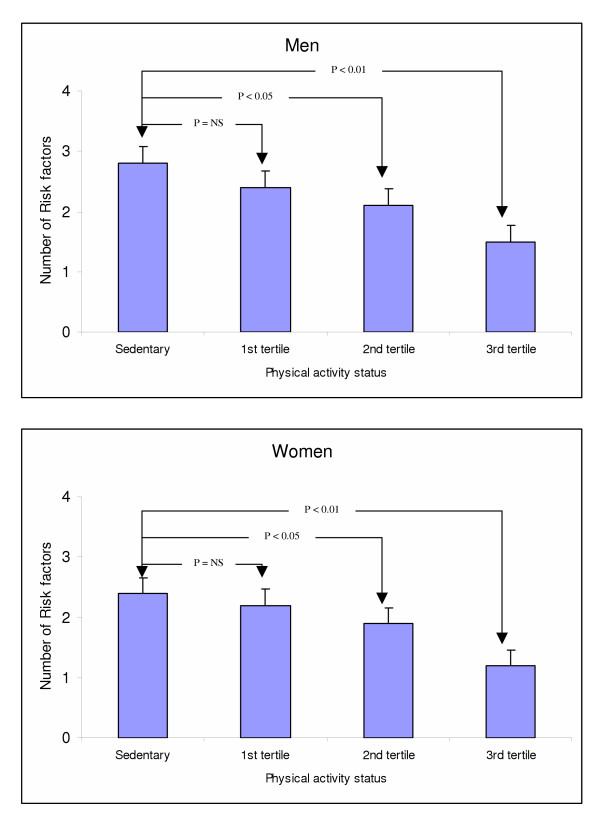
Cumulative distribution of cardiovascular risk factors score by physical activity status.

Afterwards we calculated the cumulative distribution of four major cardiovascular risk factors, i.e. obesity, hypertension, hypercholesterolemia, and diabetes. We observed an inverse relationship between physical activity index and the cumulative score of risk factors (partial *rho *= -0.33, p < 0.001), after adjusting for age, sex and smoking habits. Figure 1 illustrates the inverse association between activity status and risk factors score in men and women, respectively. Additionally, we may observed that even moderate amounts of physical activity (i.e. second tertile) have a beneficial effect on the cumulative distribution of these cardiovascular risk factors.

## Discussion

In this observational study of 3042 adult people from Attica region, Greece, we found that, roughly, one out of two men and women were physically active. Therefore, it could be speculated that, about, 4 million Greek adults are sedentary. In addition, men were more likely to be physically active as compared to women, while the lowest physical activity rates were observed in middle-aged participants (i.e. 40 to 49 years old). A description of the profile of the participants showed that physically active people had higher occupation skills, were more likely to live in rural areas, to be unmarried, to be non smokers and they were devoted to a healthier dietary pattern, as compared to sedentary, irrespective of age and sex. In addition, the cumulative score of four major cardiovascular risk factors, i.e. obesity, hypertension, hypercholesterolemia, and diabetes, was inversely associated with the applied physical activity index. Finally, physically active men and women were less likely to report depressive symptoms.

### Physical activity and socio-demographic characteristics

Physical activity has been related with social status in some, but not all studies. For example, in a population-based sample from Copenhagen [12], the investigators observed that subjects with the lowest level of education (a proxy of social status) were most frequently physically inactive, as well as heavy smokers, heavy drinkers and obese. In another community health survey of Adelaide [13] the investigators reported that low physical activity status was strongly associated with low education and reduced mobility, number of social connections and degree of satisfaction with recreation facilities. On the other hand, Tammelin et al. [14] reported that infrequent participation at young age was associated with physical inactivity at older age, while low social class of the childhood family was associated with physical inactivity in adolescence but not with inactivity at older age. In our study, we observed a positive association of physical activity with some (occupation skills), but not all social status indices (education level and annual income). This may reflect various cultural characteristics of the participants, but it could not be further investigated by the present work.

We also found that participants who were devoted in organised sports and members in sports clubs were more frequently persistent exercisers as compared to those who did not. This is in accord to the reports from several other investigators, which observed that participation in organised sports was associated with the stability of physical activity [15-17]. Thus, we may suggest that the development of programs to promote participation in organised sports it could be a worthwhile investment for their health and well-being.

We observed that men were more likely to be physically active as compared to women, irrespective of age. These gender differences are pronounced in most countries. For example, it has been reported that the proportions of girls exercising actively is approximately half of that of boys among 15-year-olds in Greenland, Lithuania and Greece [18]. The issue of gender differences is also relevant, when we consider physical activity trends from adolescents to adults. In addition, we found that the gender difference in physical fitness was even higher than the difference in physical activity status, since men were more commonly persistently fit. The aforementioned gender differences have been reported in several studies [19-21], and may reflect behavioural and work-related differences, as well as differences in family responsibilities between sexes that could not investigated in this study. However, it should be noted that Barnekow-Bergkvist et al. [22] reported that, although more men than women participated in sports activities at the age of 16, there was no significant difference at the age of 34.

Regarding marital status we observed that never married participants were more likely to be physically active as compared to married or divorced/widowed, independent from age and sex of the participants. There are very sparse data in the literature concerning the relationship between marital status and physical activity. In a population-based study of men and women from the US [23], the investigators observed that the change from a married to a single state did not affect physical activity relative to remaining married, whilst the transition from a single to a married state resulted in significant positive changes in physical activity relative to remaining single. Thus, they concluded that marriage might potentially set the stage for natural changes in physical activity status. Family, work and other social responsibilities, as well as cultural difference may be responsible for the divergence of the results between the present and other studies.

### Physical activity and depression

Depression constitutes a worldwide health problem, because is associated with functional impairment in normal life and at work, poor compliance with medical therapy and lifestyle risk factors interventions [24]. Much discussion has been made regarding the association between depression and physical activity status [25-31]. Few population-based studies reported that physical activity was not associated with depression [25-27], while other studies reported a protective effect [28-33]. For example, the results from the first National Health and Nutrition Examination Survey (NHANES) reported that the risk of depressive symptoms at follow-up were two-fold for women with little or no physical activity compared to women with much or moderate recreational physical activity [28]. Although it is difficult to compare findings from prospective with cross-sectional studies, we have found similar results with the NHANES since participants in the lowest tertile of physical activity had 1.6-times higher odds of reporting major depressive syndromes. Whether physical activity is associated with depression it is difficult to answered by the present study. Although we provided clear scientific evidence, further research is needed to state cause – effect relationships and to clarify the mechanisms underlying the association between physical activity and depression.

### Physical activity and clinical characteristics

Furthermore, we confirmed previous reports concerning an inverse association of physical activity with the prevalence of obesity and hypertension [34-36], while no relationship was observed in our, as well as several other studies, regarding physical activity and hypercholesterolemia [37,38]. We also found an inverse relationship between presence of leisure time physical activities and the cumulative distribution of four major cardiovascular risk factors (i.e. obesity, hypertension, hypercholesterolemia, and diabetes). We expanded the previous findings by showing that even moderate amounts of physical activity were associated with the frequency of these cardiovascular risk factors levels. It is becoming more apparent that most of the health benefits at a minimal risk are derived from low to moderate intensity physical activities [39].

### Limitations

This is a cross-sectional study that cannot provide causal relationships, but only state hypotheses for future research. Occupational physical activity which was not taken into account in the present study, and misreporting of physical activity status due to self-reports may confound, at least in part, the strength of the observed relationships. Another limitation is that LTPA has been related to a healthier lifestyle habits and consequently to a better health status. For example, adoption of a healthier dietary pattern or reduced smoking habits may be more common among individuals who were devoted to leisure time exercise. Thus, the benefits from LTPA on the prevalence of risk factors (Figure 1) cannot provide causal relationships and may be miss-interpreted at population level. Finally, although we took into account dietary and smoking habits of the participants the influence of the potential confounding effect of these factors cannot entirely be excluded.

## Conclusion

A considerable proportion of the population studied reported physically inactive, indicating that sedentary lifestyle becomes a serious epidemic in Greece, and especially in women. We have also illustrated the socio-demographic profile of people that are physically active and we revealed the relationships between physical activity and various lifestyle behaviors, the presence of depressive symptoms and several metabolic disorders associated with cardiovascular risk. Despite the limitations of the cross-sectional design of the present study, our findings may carry an important public health message, i.e. to inform the community and especially middle-aged, low socio-economic status people living in urban areas, about the benefits derived from physical activity in order to prevent future cardiovascular events and other psychological disorders.

## Competing interests

The author(s) declare that they have no competing interests

## Authors' contributions

CP = drafted the paper, was the principal investigator, and had the concept, and design of the study, DBP = wrote the paper, was the principal investigator, and had the concept and design of the study, performed the data analysis, YL = drafted the paper, participated in the clinical evaluation of the participants and CS = drafted the paper.

## Pre-publication history

The pre-publication history for this paper can be accessed here:


